# Decellularization of human donor aortic and pulmonary valved conduits using low concentration sodium dodecyl sulfate

**DOI:** 10.1002/term.2391

**Published:** 2017-05-12

**Authors:** Tayyebeh Vafaee, Daniel Thomas, Amisha Desai, Louise M. Jennings, Helen Berry, Paul Rooney, John Kearney, John Fisher, Eileen Ingham

**Affiliations:** ^1^ School of Biomedical Sciences, Faculty of Biological Sciences University of Leeds Leeds UK; ^2^ Institute of Medical & Biological Engineering, School of Mechanical Engineering University of Leeds UK; ^3^ The Biocentre The Biocentre Innovation Way, Heslington York UK; ^4^ Tissue & Eye Services NHS Blood & Transplant Estuary Bank Speke Liverpool UK

## Abstract

The clinical use of decellularized cardiac valve allografts is increasing. Long‐term data will be required to determine whether they outperform conventional cryopreserved allografts. Valves decellularized using different processes may show varied long‐term outcomes. It is therefore important to understand the effects of specific decellularization technologies on the characteristics of donor heart valves. Human cryopreserved aortic and pulmonary valved conduits were decellularized using hypotonic buffer, 0.1% (w/v) sodium dodecyl sulfate and nuclease digestion. The decellularized tissues were compared to cellular cryopreserved valve tissues using histology, immunohistochemistry, quantitation of total deoxyribose nucleic acid, collagen and glycosaminoglycan content, *in vitro* cytotoxicity assays, uniaxial tensile testing and subcutaneous implantation in mice. The decellularized tissues showed no histological evidence of cells or cell remnants and >97% deoxyribose nucleic acid removal in all regions (arterial wall, muscle, leaflet and junction). The decellularized tissues retained collagen IV and von Willebrand factor staining with some loss of fibronectin, laminin and chondroitin sulfate staining. There was an absence of major histocompatibility complex Class I staining in decellularized pulmonary valve tissues, with only residual staining in isolated areas of decellularized aortic valve tissues. The collagen content of the tissues was not decreased following decellularization however the glycosaminoglycan content was reduced. Only moderate changes in the maximum load to failure of the tissues were recorded postdecellularization. The decellularized tissues were noncytotoxic *in vitro*, and were biocompatible *in vivo* in a mouse subcutaneous implant model. The decellularization process will now be translated into a good manufacturing practices‐compatible process for donor cryopreserved valves with a view to future clinical use. Copyright © 2016 The Authors Tissue Engineering and Regenerative Medicine published by John Wiley & Sons, Ltd.

## 1 Introduction

Conventional heart valve replacements all have limitations. Mechanical heart valve replacements can last a life‐time; however, patients with mechanical heart valves require lifelong anticoagulation therapy. Bioprosthetic heart valves suffer from limited durability due to degeneration and calcification (Gao *et al*., 2004; Senthilnathan *et al*., 1999). Neither mechanical nor bioprosthetic heart valve replacements have the capacity for growth in children or young developing adults. Human donor cryopreserved allografts have low thrombogenicity, superior hemodynamic performance and resistance to infection; however, they fail to regenerate *in vivo* and cannot grow and develop in the recipient. They fail due to progressive degenerative changes resulting from immunologically mediated inflammation and calcification (Carr‐White *et al*., 2001; Hogan and O’Brien, 2003) and cardiac valve allografts demonstrate accelerated degeneration and the need for reoperation in children (Shaddy and Hawkins, 2002). The pulmonary autograft switch operation is considered the preferred option for children who require aortic valve replacement (da Costa *et al*., 2009; Etnel *et al*., 2016) in order to provide a living, functional aortic valve. A cryopreserved pulmonary allograft is then placed in the right ventricular outflow tract.

Since cryopreserved allogeneic heart valves contain donor cells with associated antigens that initiate an adverse host response and act as foci for calcification (Baskett *et al*., 2003; Dignan *et al*., 2003; O’Brien *et al*., 2001; Ryan *et al*., 2006; Shaddy and Hawkins, 2002), it is logical to propose that removal of the cellular components of allogeneic heart valves prior to implantation would minimize the immune response and calcification. Moreover, removal of donor cells might also encourage repopulation with endogenous cells capable of repair and remodelling, thus overcoming the limitations of degeneration over time and producing valves with the potential for growth in young patients, eliminating the need for reoperation.

Interest arose in the development of decellularized heart valve allografts at the turn of the century. An antigen reduction method process, the SynerGraft™ process was developed by CryoLife Inc (Elkins *et al*., 2001a; O’Brien *et al*., 1999) and Elkins *et al.* (2001a) were the first to report on very early clinical results of pulmonary allografts treated by the SynerGraft process. This proprietary process involves cell lysis induced by incubation in water and nuclease digestion followed by a multiday isotonic wash‐out phase (Gerson *et al*., 2012; O’Brien *et al*., 1999). Near complete removal of cells and cellular components was demonstrated through histological and immunocytochemical analysis with no corresponding change in the *in vitro* biomechanics (Elkins *et al*., 2001b). The CryoValve SG™ pulmonary valve has subsequently been reported to perform well clinically in the short to mid‐term and outcomes in adults and children with a mean follow‐up of 4 years have been comparable to (Bechtel *et al*., 2005, 2008; Brown *et al*., 2010; Burch *et al*., 2010; Hawkins *et al*., 2003) or better than (Konuma *et al*., 2009; Ruzmetov *et al*., 2012; Tavakkol *et al*., 2005) conventional cryopreserved human pulmonary allografts. The SynerGraft process has also been applied to thicker human aortic valve conduits. At 1‐year follow‐up, small numbers of the valves showed low transvalvular gradients and low panel specific antibody responses (Zehr *et al*., 2005).

Decellularized human donor valve allografts produced using different processes have also shown promise in clinical studies despite a lack of detailed description of the biological and biomechanical characteristics of the valves in the literature. da Costa et al. (2009) reported on good results in the Ross procedure (up to 4 years)using pulmonary valves decellularized using low concentration sodium dodecyl sulfate (SDS). Clinical studies of decellularized aortic valve allografts showed stable structural integrity, low rate of calcification and adequate haemodynamics in the short to medium term (da Costa *et al*., 2010). Up to 6 years’ follow‐up, SDS decellularized pulmonary allografts were associated with greater freedom from reoperations compared to conventional cryopreserved allografts (da Costa *et al*., 2014). In Germany, the Haverich group have reported on the early results of fresh pulmonary allografts decellularized using 0.5% (*w*/*v*) sodium deoxycholate and 0.5% (*w*/*v*) SDS in children and young adults (Cebotari *et al*., 2011; Neumann *et al*., 2014). In comparison to glutaraldehyde‐fixed bovine jugular vein and cryopreserved allografts, the decellularized fresh allografts showed improved performance (Cebotari *et al*., 2011).

These studies indicate that decellularization processes do not affect the *in vivo* performance of allogeneic heart valves; however, longer‐term data will be required to determine whether they out‐perform conventional cryopreserved allografts and reduce or indeed eliminate the need for reoperation. Importantly, since the decellularized allografts that have been used clinically have been produced using different processes, the longer‐term outcome may differ dependent upon the specific decellularization process. It is therefore important to understand the effects of specific decellularization processes on the characteristics of allogeneic heart valves.

In the UK, conventional cryopreserved human donor valves are processed, stored and supplied to surgeons through NHS Blood & Transplant Tissue & Eye Services (NHS BT TES). With a view to improving clinical performance, we have collaborated in the development of robust decellularization processes for human donor heart valves. A process for the decellularization of porcine aortic and pulmonary roots was initially developed and the biological and biomechanical characteristics of these valves have been described (Booth *et al*., 2002; Korossis *et al*., 2005; Luo *et al*., 2014; Wilcox *et al*., 2005). Decellularized porcine aortic roots were shown to perform well and demonstrated regenerative potential in the right ventricular outflow tract of juvenile sheep over a 6‐month period (Paniagua Gutierrez *et al*., 2015). We have subsequently applied our proprietary process to human donor aortic and pulmonary valved conduits and here we present a fully comprehensive description of the process and biological characteristics of the decellularized valved conduits.

## 2 Materials and methods

### 2.1 Human donor cryopreserved cardiac valves

The human donor aortic and pulmonary cardiac valves used in this study were supplied by NHS BT TES, Speke, Liverpool. The valves were found to be unsuitable for therapeutic use but had full research and development consent. Valves were cryopreserved using the standard NHS BT TES process and stored at –80°C prior to use. The study was conducted under UK NHS Health Research Authority approval (REC 09/H1307/02). In total, 26 aortic {14 male, 40 ± 9 years [± 95% confidence limits (CL)]; 12 female, 46 ± 8 years} and 26 pulmonary (14 male, 47 ± 11 years; 12 female; 48 ± 11 years) donated cardiac valves were used during the course of these studies.

### 2.2 Decellularization process

Cryopreserved valved conduits were decellularized in batches of four or six (half aortic; half pulmonary). All steps of the process were carried out aseptically in a class II microbiological safety cabinet. The bags containing human cryopreserved valves were submerged in a water bath at 37°C with gentle agitation for 9–12 min. The outer and inner bags were cut open and the contents were placed into a sterile 2‐l beaker. The cryopreservation solution was diluted by the sequential addition of 200, 400 and 800 ml phosphate‐buffered saline wash solution [PBS plus 2.7 mm diaminoethane tetra‐acetic acid disodium salt (EDTA; VWR) and 10 KIU/ml aprotinin (Nordic Pharma)] after 5, 10 and 15 min. The valves were placed in fresh (200 ml) wash solution and left for 5 min before scraping the adventitial layer of the valves with a scalpel. Each valve was then sutured through the ventricular muscle and the distal arterial wall to the base and neck of a 500‐ml wide‐necked polypropylene bottle (Thermo Fisher Scientific Ltd) using a 2.0 TI suture (Syneture) to ensure that the valve lumen remained open during decellularization. Subsequent wash steps were carried out using 8 ml/g solution at 40–45°C with agitation [240 rotations/min (rpm)] with the bottles laid horizontally unless otherwise stated. Each valve was washed with three changes of PBS wash solution for 5–15 min; hypotonic Tris buffer [10 mm Tris (VWR), 0.1% (*v*/v) EDTA, 10 KIU/l aprotinin, pH 8.0–8.2] for 24 h; 0.1% (*v*/v) SDS (Sigma–Aldridge) in hypotonic Tris buffer for 24 h; PBS plus 10 KIU/l aprotinin, for 2 × 5–15 min and then 24 h. The valves were then incubated in nuclease solution (2–4 ml/g at 80 rpm) for 3 h at 37°C. This step was repeated. In initial experiments the nuclease solution comprised 50 U/ml deoxyribose nuclease (Pulmozyme, Roche), 1 U/ml RNAse (Ribonuclease T1; Worthington), 10 mm MgCl_2_ in 50 mm Tris pH 7.5. This was subsequently replaced with 10 U/ml Benzonase (Novagen) in 50 mm Tris, 1 mm MgCl_2_, pH 8.0–8.2.

The valves were then washed in PBS for 5–15 min (twice) and 24 h, disinfected in Cambridge antibiotic solution (Source BioScience; 80 rpm) for 3 h at 37°C, washed again in PBS for 66 h and then in hypertonic buffer (50 mm Tris, 1.5 m NaCl, pH 8.0–8.2) for 24 h and PBS for 5–15 min (twice) and 24 h. Valves were stored at 4°C in PBS and used for analyses within 1 week or cryopreserved for biomechanical evaluation.

All decellularized conduits were subject to histology. Initial batches produced using Pulmozyme as the nuclease were analysed for total deoxyribose nucleic acid (DNA), collagen and glycosaminoglycan (GAG) content plus extract cytotoxicity tests. Subsequent batches were produced using Benzonase as the nuclease and these were used for contact cytotoxicity tests, immunohistochemistry, *in vivo* biocompatibility in mice and biomechanical analyses.

### 2.3 Histology and immunohistochemistry

Samples of cellular and decellularized valves were taken longitudinally to incorporate half a leaflet, the junctional region, artery wall and myocardial skirt, placed in histology cassettes and fixed in 10% (*v*/v) neutral buffered formalin for 24 h, dehydrated and embedded in paraffin wax. Serial sections (10 μm) were cut longitudinally at two levels 120 μm apart. Sections from each level were stained with haematoxylin and eosin (H&E) using standard methods. Sections at each level were also stained using 4′,6‐diamidino‐2‐phenylindole dihydrochloride (DAPI; Sigma–Aldrich). The sections were rehydrated and immersed in DAPI solution (1 mg/ml in 10 mm Tris, 1 mm Na_2_EDTA, 1 mm NaCl pH 7.4) for 10 min, washed in PBS and mounted in fluorescence mounting medium (Dako) to detect residual double stranded DNA in the tissues. Von Kossa staining of sections was used to visualize calcium and performed as follows. Sections were immersed in 1% (*w*/*v*) silver nitrate (Sigma) for 1 h on a light box and then washed in sodium thiosulfate (158 mm; VWR) for 5 min, washed in distilled water and then counterstained in fast red (0.1% *w*/*v* nuclear fast red (Sigma) 5% *w*/*v* aluminium sulfate (Thermo Fisher).

Immunolabelling was carried out using an Envision detection system (Thermo Scientific). Rabbit polyclonal antibodies to von Willebrand factor (VWF; 1:200; Dako A0082) and fibronectin (1:100; Dako A0245) and mouse monoclonal antibodies to chondroitin sulfate (1:200; Sigma CS‐56 IgM), collagen IV (1:50; Dako CIV22 IgG_1_), laminin (1:800; Sigma LAM‐89; IgG_1_) and rabbit monoclonal to HLA‐A (1:30; Abcam EP1395Y) were used to determine the retention/removal of extracellular matrix (ECM) and membrane proteins following decellularization. For detection of VWF and collagen IV, microwave antigen retrieval was used (10 mm citric acid pH 6.0 for 10 min). For detection of fibronectin, chondroitin sulfate and laminin, trypsin antigen retrieval was carried out by incubating in 0.1% (*w*/*v*) trypsin in 0.1% (*w*/*v*) CaCl_2_ at a pH of 7.8 for 10 min at 37°C. Appropriate isotype control antibodies and omission of the primary antibodies served as a negative controls.

Images were captured using an upright Olympus BX51 light and fluorescence microscope fitted with a DAPI filter, Olympus XC50 digital camera and Cell B software (Olympus®, UK).

### 2.4 Total DNA extraction and quantification

DNA was extracted from four areas of cellular and decellularized aortic and pulmonary roots (*n =* 3 or 4; leaflet, artery wall, myocardial skirt and the junction of the leaflet to the wall) using a QIAamp DNA mini kit (Qiagen). DNA was extracted from 25 mg of cellular tissues and 80–250 mg of decellularized tissues. Extracted DNA was quantified using a nanodrop spectrophotometer at 260 nm (Thermo Fisher Scientific).

### 2.5 Biochemical assays

Triplicate samples of the arterial wall tissue (~200 mg) and one leaflet (~25 mg) from three cellular and three decellularized aortic and pulmonary roots were finely macerated and lyophilized to constant weight.

#### 2.5.1 Hydroxyproline assay

Hydroxyproline content was used as a measure of total collagen content. Lyophilized samples were hydrolyzed in 6 m HCl for 4 h at 120°C and 103.4 KPa. The hydrolyzed solutions were neutralized with 6 m NaOH. Diluted samples of the hydrolyzed tissue samples were assayed as described by Luo *et al*. (2014). The hydroxyproline content of the samples was determined by interpolation from a standard curve of trans‐4‐hydroxy‐L‐proline concentration against absorbance at 570 nm.

#### 2.5.2 GAG assay

Lyophilized samples were incubated with 5 ml of papain (50 U/ml (Sigma); in PBS plus 5 mm cysteine‐HCl (Sigma) and 5 mm EDTA; pH 6.0) for 42 h at 60°C. Digested tissue samples (40 μl) were mixed with 250 μl 1,9 dimethylene blue (Sigma) dye solution [1.6% (*w*/*v*) in 0.5% (*v*/v) ethanol (VWR), 0.2% (*v*/v) formic acid (Sigma), 0.2% (*w*/*v*) sodium formate (VWR); pH 3.0]. Optical densities were determined after 1 min using a microplate spectrophotometer at 525 nm. The GAG content was measured by interpolation from a standard curve of chondroitin sulfate B concentration against absorbance.

### 2.6 *In vitro* cytotoxicity assays

Extract and contact cytotoxicity tests were carried out using 3 T3 murine fibroblasts and baby hamster kidney (BHK) cells (BHK 21 Strain 31, ECACC). 3 T3 cells were cultured in Dulbecco's modified Eagle's medium (MEM; Sigma) plus 10% (*v*/v) fetal bovine serum (Sera Lab), 100 U/ml penicillin, 100 μg/ml streptomycin (Sigma), and 2 mm L‐glutamine (Sigma). BHK cells were cultured in Glasgow's MEM (Sigma) containing 5% (*v*/v) FBS, 2.5% (*v*/v) tryptose phosphate broth (Oxoid) plus 100 U/ml penicillin, 100 g/ml streptomycin, 2 mm L‐glutamine at 37°C in 5% (*v*/v) CO_2_ in air.

For extract tests, triplicate samples of macerated decellularized aortic (*n =* 4) and pulmonary wall tissues (*n =* 4) were incubated in Dulbecco's MEM or Glasgow's MEM (100 mg tissue per ml) for 72 h with agitation and centrifuged at 600 × *g* for 15 min. The supernatants were checked for sterility prior to addition to cell cultures. 3 T3 (1.25 × 10^5^/ml) and BHK (5 × 10^4^/ml) cells were seeded (200 μl) into 96‐well plates and incubated for 16 hours at 37°C in 5% (*v*/v) CO_2_ in air. The culture medium was removed and replaced with 100 μl of fresh medium with double concentration supplements plus 100 μl of the tissue extract. The controls comprised 200 μl of 40% (*v*/v) dimethyl sulfoxide (DMSO; Sigma) in culture medium (positive) or culture medium alone (negative). The cells were cultured for 48 h and the adenosine triphosphate (ATP) content of the cells determined using the ATP Lite‐M® assay (Packard) according to the manufacturer's instructions. Data were recorded as luminescent counts/s.

For the contact assay, triplicate samples of decellularized aortic and pulmonary wall (4–5 × 2–3 mm; *n =* 3) were attached to the bottoms of six‐well culture plates using collagen gel (extracted from rat tails). 3 T3 or BHK cells (2 ml) were seeded into each well at 2 × 10^5^/ml. Cyanoacrylate glue was used as the positive control, collagen gel and cells alone were used as the negative controls. Plates were incubated at 37°C in 5% (*v*/v) CO_2_ in air for 48–72 h (approximately 80% confluence). The plates were then viewed by phase contrast microscopy to assess whether the cells grew up to and in contact with the tissue with normal cellular morphology.

### 2.7 Subcutaneous implantation in mice

The response to cellular and decellularized aortic and pulmonary wall and leaflet tissues was assessed using a mouse subcutaneous implant model. Samples of tissues (3 × 3 mm; 1 per mouse) were implanted subcutaneously along the mid‐dorsal line of C3H mice (female; 4–6 weeks old; Harlan). Mice were fed food and water *ab libitum*, monitored and weighed weekly for a period of 3 months. Mice were then humanely sacrificed, and the implanted tissues retrieved for analysis. All animal procedures were carried out under appropriate UK Home Office licenses.

The explanted tissues together with the surrounding skin were fixed in 10% (*v*/v) neutral buffered formalin for 24 h, processed into paraffin wax blocks and H&E stained tissue sections were used to determine cell infiltration and morphology. The sections were viewed and scored by two independent observers. The thickness of the capsule surrounding the explanted tissue was determined by taking six measurements (Image Pro Plus® imaging software; Media Cybernetics) at predetermined locations along the circumference of the tissue explants. The cellular response to the implanted tissues was assessed at six predetermined locations around the periphery of the implants and the number of polymorphonuclear cells (PMNs), mononuclear phagocytes and lymphocytes per 40× field of view assessed using a scale of 0–4 (0 = no cells; 1 = 1–5 cells; 2 = 5–10 cells; 3 = heavy; 4 = packed). The total number of giant cells was recorded and the overall degree of cellular infiltration into the implants was assessed alongside the extent of calcification and necrosis as slight, moderate, marked or heavy.

### 2.8 Uniaxial tensile tests

Uniaxial tensile tests were conducted on cellular cryopreserved aortic (*n =* 8) and pulmonary (*n =* 4) valve and decellularized aortic (*n =* 4) and pulmonary (*n =* 4) valve tissues from conduits that had been cryopreserved again postdecellularization. This was to mimic the means by which the conduits would be preserved postdecellularization. Root wall and leaflet specimens from all the cellular and decellularized valves were subjected to uniaxial tensile testing to failure using an Instron 3365 (Instron® Corporation) fitted with a 50 N load cell at 10 mm/min under hydrated conditions in PBS at 37°C. Tissue specimens with 10 mm gauge length and 5 mm width were prepared from the valve root wall in the axial and circumferential directions from each valve, and from the valve leaflet in the circumferential direction. Due to size limitations, the radial specimens from valve leaflets were 3 mm in width with a 6 mm gauge length. Each tissue specimen thickness was measured with a digital thickness gauge J‐40 V, having a precision of 0.01 mm. The measured load was plotted against the extension and maximum load was recorded for all tissue specimens.

### 2.9 Data analysis

The Student *t* test was used for comparison of groups of two means and one‐way analysis of variance (anova) was used for the comparison of data from more than two groups. Following anova, individual differences between group means were calculated using the T‐method to determine the minimal significant difference (*p* < 0.05).

## 3 Results

### 3.1 Histological and immunohistochemical assessment of cellular and decellularized tissues

**Figure 1:**
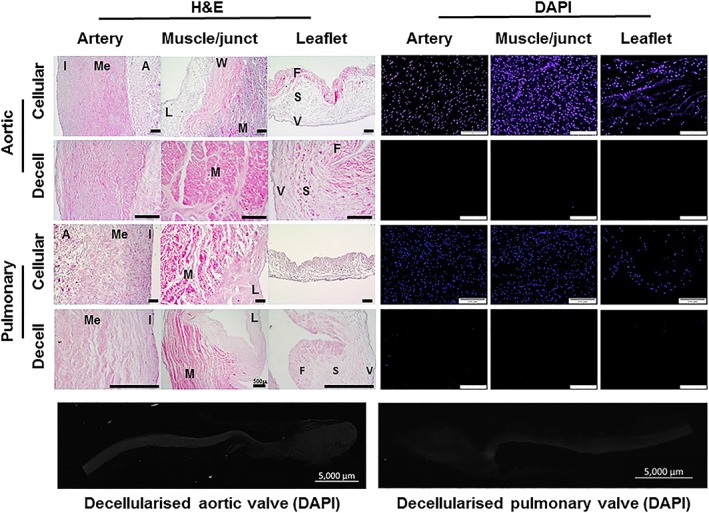
**Images of H&E and DAPI stained sections of cellular cryopreserved and decellularized aortic and pulmonary valved conduit tissues.** H&E‐stained sections of the cellular arterial wall components of the aortic and pulmonary valved conduits show the cellular distribution in the intimal (I), medial (Me) and adventitial (A) regions. The junctional region is the area where the arterial wall (W), muscle (M) and leaflet (L) merge. The trilaminar structure of the cellular leaflet tissues is clearly visible with the fibrosa (F), spongiosa (S) and ventricularis (V) layers in the aortic and pulmonary leaflets. DAPI stained sections of the cellular tissues show the cell distribution in the arterial wall, muscle and leaflets, with sparser cells in the leaflet tissues. Sections of the decellularized aortic and pulmonary valve tissues show no evidence of cells or cell nuclei when stained with H&E or DAPI and show that the histoarchitecture of both the aortic and pulmonary valve tissues is well preserved following decellularization. Scale bars are 200 μm unless otherwise labelled [Colour figure can be viewed at wileyonlinelibrary.com]

H&E‐stained sections of cellular tissues showed the cell distribution throughout the aortic and pulmonary root (Figure 1). The media layer of the aortic arterial wall was markedly thicker than that of the pulmonary artery wall, which appeared less organized (Figure 1). The junctional region is the area of the tissue where the root of the valve leaflet attaches to the ventricular muscle and arterial wall as illustrated for the aortic valve in Figure 1. DAPI stained sections of the cellular tissues showed the distribution of cell nuclei within the arterial wall and muscle/ junction regions with sparser numbers of cell nuclei within the cellular leaflet tissues (Figure 1).

Sections of the decellularized aortic and pulmonary valve tissues showed no evidence of cells or cell nuclei when stained with H&E. Representative sections of the aortic valve arterial wall, ventricular muscle and leaflet (Figure 1) together with images of sections of the pulmonary valve arterial wall, junctional region and leaflet (Figure 1) showed that the histoarchitecture of both the aortic and pulmonary valves was well preserved following decellularization. DAPI stained sections showed no evidence of cell nuclei within the decellularized tissues (Figure 1).

**Figure 2:**
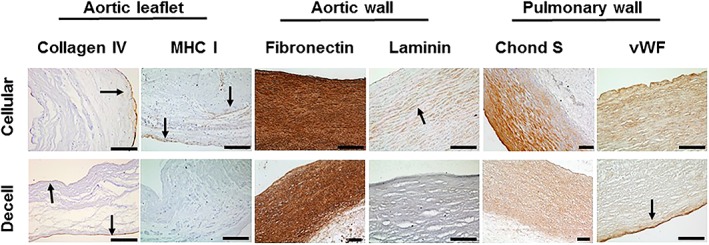
**Immunohistochemical analysis of sections of cellular cryopreserved and decellularized aortic and pulmonary valved conduit tissues.** Images of sections of cellular and decellularized aortic valve leaflet tissue stained with antibodies to collagen IV and MHC Class I show retention of collagen IV in the intimal basement membrane (arrows) and loss of MHC Class I immunostaining following decellularization. Images of sections of cellular and decellularized aortic wall tissue stained with antibodies to fibronectin and laminin show a reduction of fibronectin and laminin (both cell adhesion proteins) postdecellularization. Images of sections of cellular and decellularized pulmonary wall tissues show a reduction in chondroitin sulfate (Chond S; representative of GAGs) and VWF following decellularization. Scale bars 200 μm unless otherwise labelled [Colour figure can be viewed at wileyonlinelibrary.com]

Sections of cellular and decellularized aortic and pulmonary valve tissues were stained with antibodies to extracellular matrix proteins and representative images are shown in Figure 2. The degree of staining was qualitatively graded on a scale of 0–5 in the arterial wall and leaflet tissues and the results are summarized in Table 1. There was good retention of collagen IV and VWF staining in the basement membrane of the tissues following decellularization and some loss of intensity of fibronectin, laminin and chondroitin sulfate staining in the tissues following decellularization.

**Table 1:** **Qualitative assessment of immunohistochemical staining of sections of cellular and decellularized aortic and pulmonary valve tissues.** Sections of tissues stained with antibodies to the extracellular matrix components were graded on a scale of – (no staining) to +++++ (intense staining). Collagen IV was graded in the matrix plus basement membrane of the wall tissues and the basement membrane of the leaflets, according to where it was expressed. MHC Class 1 was graded on cells in cellular tissue. A grade of ± in the decellularized tissue indicates isolated residual staining

		**Collagen IV**	**Fibronectin**	**VWF**	**Laminin**	**Chondroitin sulfate**	**MHC Class I**
Aortic valve							
Wall	Cellular	**+++**	**+++++**	**++**	**++**	**+++**	**++**
	Decelled	**++**	**++++**	**++**	**+**	**++**	**±**
Leaflet	Cellular	**++**	**+++**	**+**	**+**	**++**	**++**
	Decelled	**++**	**+++**	**+**	**±**	**++**	**±**
Pulmonary valve							
Wall	Cellular	**+++**	**++++**	**++**	**+**	**+++**	**++**
	Decelled	**++**	**++**	**++**	**±**	**++**	**–**
Leaflet	Cellular	**++**	**++**	**+**	**+**	**+++**	**++**
	Decelled	**++**	**++**	**+**	**ND**	**++**	**–**

ND = not done.

Immunohistochemical staining for major histocompatibility complex (MHC) Class I showed expression of MHC Class I by the majority of cells in the cellular tissues (Figure 2). Following decellularization there was an absence of MHC Class I staining in the pulmonary valve arterial wall and leaflet tissues, with some evidence of residual staining in isolated areas of the decellularized aortic wall and leaflet tissues (Figure 2; Table 1).

### 3.2 Total DNA content

Total DNA content (per wet tissue weight) of the cellular and decellularized tissues is presented in Table 2. The DNA content of cellular valve tissues ranged from 211 ng/mg (pulmonary valve junction) to 618 ng/mg (aortic valve ventricular muscle). The DNA content of the cellular valve tissues showed a high degree of variation, however, overall the DNA content was greatest in the ventricular muscle and lowest in the leaflets and junctional regions. Following decellularization the DNA content was <10 ng/mg for all tissues except for the pulmonary leaflet (19.7 ng/mg). For all other decellularized tissues, the DNA content was reduced to <3% of the cellular tissues.

**Table 2:** **Total DNA in cellular and decellularized aortic and pulmonary valve tissues and percentage remaining after decellularization.** The total DNA was extracted from three samples of tissues from each of four areas (leaflet, wall, junction and muscle) of three cellular aortic, four decellularized aortic, three cellular pulmonary and four decellularized pulmonary valves and quantified by nanodrop spectrometry

		Total DNA ng/mg ± 95% CL		% DNA in decellularized			Total DNA ng/mg ± 95% CL		% DNA in decellularized
		Cellular	Decellularized				Cellular	Decellularized	
**Aortic**	**Leaflet**	231 ± 246	5.9 ± 4.8*	2.5%	**Pulmonary**	**Leaflet**	298 ± 321	19.7 ± 16.6*	6.5%
	**Wall**	294 ± 268	3.0 ± 0.9*	1.0%		**Wall**	316 ± 226	2.3 ± 0.9*	0.7%
	**Junction**	214 ± 259	2.1 ± 2.2*	1.0%		**Junction**	211 ± 248	6.1 ± 0.7*	2.9%
	**Muscle**	618 ± 1253	2.1 ± 0.9	0.3%		**Muscle**	460 ± 333	3.4 ± 5.4*	0.7%

The total DNA content is presented as the mean (*n =* 3 or 4) ± 95% CL. The data for each type of tissue before and after decellularization was analysed by Student *t* test, which revealed a significant reduction in DNA levels for all tissues (*p < 0.05) with the exception of the aortic valve muscle tissue (*p* = 0.08). The percentage DNA remaining after decellularization was determined from the mean values for each region of tissue.

### 3.3 Hydroxyproline and GAG content

The total collagen content of the cellular and decellularized cardiac valve arterial wall and leaflet tissues (Table 3) was assessed by determination of the hydroxyproline content and then multiplying the value by 7.14 (Neuman and Logan, 1950). The collagen content of the arterial wall of the cellular aortic and pulmonary valves was similar (324 and 319 μg/mg respectively) and this did not change significantly following decellularization. The collagen content of the cellular aortic and pulmonary valve leaflets was higher (511 and 554 μg/mg respectively) and the collagen content of both types of leaflet tissue increased significantly following decellularization (736 and 765 μg/mg respectively). The GAG content of the cellular aortic valve tissues (Table 3; 10.5 μg/mg wall; 11.4 μg/mg leaflet) was slightly greater than the GAG content of the pulmonary valve tissues (6.6 μg/mg wall; 8 μg/mg leaflet). Following decellularization, the GAG content of the conduit wall tissues was reduced by 34% (aortic) and 40% (pulmonary), although this was only significant for the pulmonary artery tissues (Student *t* test; *p* = 0.008). There was a significant and marked decrease of 75% (aortic) and 74% (pulmonary) in the GAG content of the decellularized valve leaflets compared to cellular tissues (Table 3).

**Table 3:** Total collagen and glycosaminoglycan content of cellular and decellularized aortic and pulmonary valve tissues. The total collagen and glycosaminoglycan (GAG) content of the arterial wall and leaflet tissues of three cellular aortic and pulmonary valves and three decellularized aortic and pulmonary valves was quantified

		**Cellular** μg**/mg**	**Decellularized** μg**/mg**	***p***
**Collagen**				
**Aortic valve**	Wall	323.59 ± 112.23	402.28 ± 115.89	0.104
	Leaflet	511.15 ± 186.2	735.69 ± 254.68	0.038*
**Pulmonary valve**	Wall	318.70 ± 118.82	398.76 ± 107.86	0.099
	Leaflet	553.57 ± 190.32	764.71 ± 90.22	0.013*
**GAGs**				
**Aortic valve**	Wall	10.49 ± 6.56	6.93 ± 5.4	0.145
	Leaflet	11.36 ± 0.39	3.94 ± 3.1	0.001*
**Pulmonary valve**	Wall	6.57 ± 2.21	3.93 ± 0.6	0.008*
	Leaflet	7.98 ± 2.83	2.89 ± 1.5	0.002*

The total collagen and GAG content are presented as the mean (*n =* 3) ± 95% CL. The data for each tissue type before and after decellularization were analysed using the Student *t* test, which revealed a significant increase in collagen content of decellularized aortic and pulmonary valve leaflets and a significant decrease in GAG content of aortic valve leaflets and pulmonary valve leaflets and wall tissue following decellularization.

### 3.4 *In vitro* cytotoxicity testing

**Figure 3:**
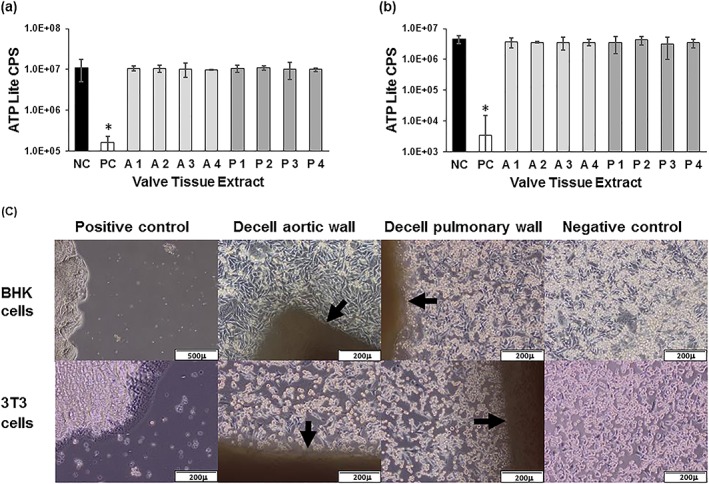
**Extract and contact cytotoxicity assays of decellularized aortic and pulmonary artery wall tissues.** ATP levels of BHK cells (a) and 3 T3 cells (b) following 48 h culture with decellularized tissue extracts. Data are presented as the mean (*n =* 3) luminescent counts/s (CPS) ± 95% CL. NC negative control (cells with no extract); PC positive control (40% DMSO); A1–A4 aortic wall extracts; P1–P4 pulmonary wall extracts. Data were analysed by one‐way analysis of variance for each cell line. This showed no significant difference in the mean ATP levels in the presence of the extracts compared to cells cultured in medium alone. There was a significant difference (* *p* < 0.05) in the ATP levels of the positive control (DMSO) compared to all other groups for both 3 T3 and BHK cells. (c) Phase contrast microscopy images of BHK cells (top panel) and 3 T3 cells (bottom panel) growing in contact with aortic wall tissue (AW) and pulmonary wall tissue (PW) but not the positive control (PC) of cyanoacrylate. NC cells cultured alone [Colour figure can be viewed at wileyonlinelibrary.com]

In order to determine whether the decellularized tissues were cytocompatible, extract and contact cytotoxicity tests were carried out using arterial wall tissues (Figure 3). There were no significant difference between the ATP levels of BHK (Figure 3a) or 3 T3 cells (Figure 3b) cultured for 48 h in the presence of the decellularized tissue extracts compared to the cell lines cultured in medium alone (anova). The positive control of 40% DMSO significantly reduced (*p* < 0.05; anova) the ATP content of both 3 T3 and BHK cells following 48 h of culture. Similarly, the decellularized tissues showed no evidence of toxicity when cultured in contact with 3 T3 or BHK cells (Figure 3c). The cells grew up to and in contact with the tissues and showed no evidence of change in morphology compared to the cells cultured alone on tissue culture plastic. The positive control of cyanoacrylate clearly killed the cells in close proximity in culture.

### 3.5 *In vivo* biocompatibility

**Figure 4:**
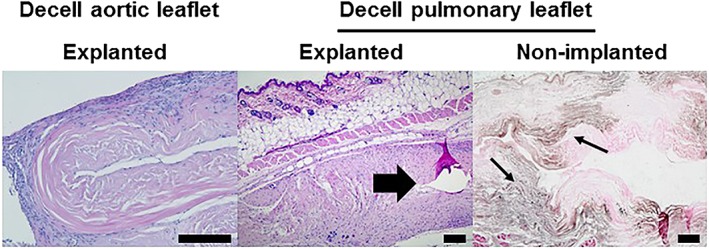
**Images of sections of explanted decellularized aortic and pulmonary valve leaflets from initial *in vivo* study in mice stained with H&E and von Kossa stained image of nonimplanted pulmonary valve tissue.** Samples of decellularized tissues were implanted subcutaneously in mice for 12 weeks. The explanted acellular aortic leaflet tissue showed good biocompatibility and integration. The explanted decellularized pulmonary leaflet tissue showed good biocompatibility and integration with a focal area of calcification (arrow). Von Kossa staining of sections of non‐implanted decellularized pulmonary leaflet tissue showed the presence of calcium (arrows). Scale bars 200 μm [Colour figure can be viewed at wileyonlinelibrary.com]

In initial studies, samples of cellular and decellularized aortic and pulmonary valve leaflet and wall tissues, each from three donors were implanted in mice and left *in situ* for 12 weeks. Upon explantation, the tissues were analysed using histology. Despite evidence that the decellularized tissues had integrated and had been recolonized by host cells, there was evidence of calcification in a proportion of the decellularized explanted tissue samples (Figure 4). A review of age of donors of these decellularized valves [mean ± standard deviation (SD) 58 ± 6 years; 3 females] and retrospective analysis of remaining samples of the implanted decellularized tissues by von Kossa staining (Figure 4) suggested that residual calcium in the decellularized valve tissues exacerbated this reaction.

**Figure 5:**
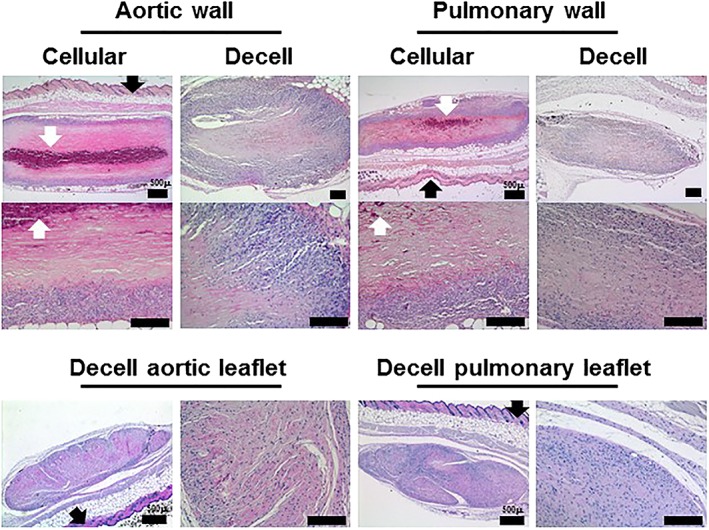
**Images of sections of explanted cellular and decellularized aortic and pulmonary valve tissues (stained with H&E) 12 weeks after subcutaneous implantation in mice.** Samples of cellular and decellularized aortic and pulmonary artery tissues from the same valved conduits were implanted subcutaneously in mice for 12 weeks. Both the cellular aortic and pulmonary artery show evidence of capsule formation, limited cell infiltration and areas of calcification and tissue deterioration. The explanted decellularized aortic and pulmonary arterial wall tissues show high numbers of cells infiltrating the peripheral regions of the implants with cells penetrating the central regions and little evidence of tissue calcification or deterioration. Samples of decellularized pulmonary and aortic valve leaflet tissues implanted subcutaneously in mice for 12 weeks were fully infiltrated with cells with no evidence of tissue deterioration or calcification. Black arrows indicate mouse epidermis. White arrows indicate areas of calcification. Occasional black spots are sectioned mouse hair. Scale bars 200 μm unless otherwise labelled [Colour figure can be viewed at wileyonlinelibrary.com]

The study was repeated using valves from donors of a younger age. Four pulmonary valves (mean ± SD age 26 ± 8 years; three male, one female) and two aortic valves (35 and 36 years; female) were selected. Prior to decellularization, a sample of the distal portion of the arterial wall of each valve was taken and divided into two for (a) implantation in mice (cellular tissue) and (b) histological evaluation with H&E and von Kossa staining. The remainder of each valve was then decellularized, dissected longitudinally into three portions (including wall, leaflet and ventricular muscle). One portion was subject to histological evaluation and samples of the remaining tissues were implanted in mice. Histological staining of the cellular valve arterial wall prior to decellularization revealed spots of calcification in the aortic valve wall tissues, with fewer spots in the pulmonary wall tissues. Following decellularization there was no evidence of any remaining calcium deposits in the wall or leaflet tissues and the histology confirmed successful decellularization. Samples of the cellular arterial wall, decellularized arterial wall and decellularized leaflet tissues (*n =* 4; 1 per donor valve for pulmonary and 2, one from each remaining portion of donor valve for aortic) were implanted into mice and left *in situ* for 12 weeks. Upon explantation the tissues were analysed using histology. The results are presented in Figure (5).

The explanted cellular aortic and pulmonary arterial wall tissues were surrounded by a capsule of cells which were predominantly macrophages with only occasional lymphocytes, PMNs and giant cells. Very few cells had infiltrated into the central areas of the implanted tissues, which showed areas of calcification and tissue deterioration (Figure 5). In contrast the explanted decellularized aortic and pulmonary arterial wall tissues were not surrounded by thickened capsules of cells, but rather high densities of cells infiltrated the peripheral regions of the implants (Figure 5). Moreover, cells were observed penetrating the central regions of the implanted decellularized wall tissues. The cells in the peripheral regions of the implants were predominantly macrophages with only occasional lymphocytes, PMNs and giant cells. There was little evidence of tissue calcification or deterioration. The decellularized pulmonary and aortic valve leaflet tissues were biocompatible in the mouse model (Figure 5) with little evidence of fibrous capsule development and cells had infiltrated into the central areas of the implants. Again, the cells in the peripheral regions of the implants were predominantly macrophages with only occasional lymphocytes, PMNs and giant cells.

**Figure 6:**
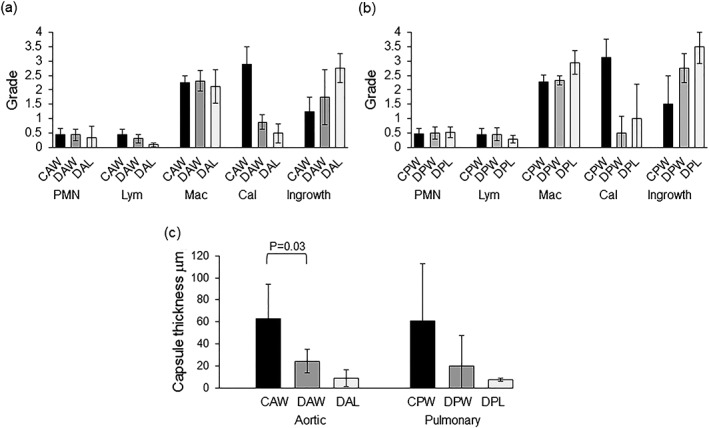
**Semiquantitative histopathological analysis of explanted cellular and decellularized aortic and pulmonary valve tissues following 12 weeks subcutaneous implantation in mice.** (a) Semiquantitative analysis of aortic valve tissues (b) Semiquantitative analysis of pulmonary valve tissues. Numbers of polymorphonuclear leucocytes (PMNs), lymphocytes (Lym) and macrophages (Mac) were graded on a scale of 0–4 where 0 (no cells); 1 (rare cells 1–5); 2 (5–10 cells); 3 (heavy cells) and 4 (packed cells) per 1600 μm^2^. Cellular ingrowth and calcification were graded on a scale 0–4 where 0 (none); 1 (slight); 2 (marked), 3 (moderate) and 4 (severe) over the whole of the explant. Data are presented as the mean (*n =* 4 explants) ± SD. (c) Average thickness of capsule surrounding explanted tissues. Data are presented as the mean (*n =* 4 explants) ± SD. Data for cellular and decellularized wall explants were compared using Student *t* test which revealed a significant difference between explanted cellular and decellularized aortic wall tissue but not explanted cellular and decellularized pulmonary wall tissue. CAW, cellular aortic wall; DAW, decellularized aortic wall; DAL, decellularized aortic leaflet; CPW, cellular pulmonary wall; DPW, decellularized pulmonary wall; DPL; decellularized pulmonary leaflet

The semiquantitative histopathological analysis of the implanted tissues is presented in Figure 6. This analysis revealed no obvious differences in the nature of the cellular infiltrate in the peripheral regions of the implanted cellular vs. decellularized tissues. There were, however, marked differences in the grades for calcification and cellular infiltration into the central regions of the implants. The cellular arterial wall tissues showed marked calcification and slight cellular ingrowth whilst the matched decellularized arterial wall tissues showed no to slight calcification and moderate to marked cellular ingrowth. The capsule surrounding the cellular arterial wall tissues was notably thicker than the capsule surrounding the decellularized arterial wall tissues although this was only significant (*p* = 0.03; Student *t* test) for the aortic wall tissue. The decellularized leaflet tissues showed marked cellular ingrowth with an absence of overt calcification and capsule formation.

### 3.6 Biomechanical assessment of decellularized tissues

The maximum load for the cellular and decellularized aortic and pulmonary valve tissues was determined from the uniaxial tensile tests and is detailed in Table 4. There was no significant difference (*p* > 0.05) in the mean maximum load between the cellular and decellularized tissues for axial and circumferential aortic wall specimens, radial aortic leaflet specimens, and circumferential pulmonary wall and leaflet specimens. However, a significant increase in mean maximum load for the decellularized aortic leaflet specimens in the circumferential direction was observed (*p* < 0.05). Also the mean maximum load for the axial decellularized pulmonary wall specimens and the radial decellularized pulmonary leaflet specimens was significantly reduced (*p* < 0.05).

**Table 4:** Mean maximum load of cardiac valve tissues before and after decellularization

**Tissue specimens**		**Number of samples tested**	**Mean maximum load ± 95% CL**
**Axial wall samples**	Cellular aortic	8	6.91 ± 1.95
	Decellularized aortic	4	8.31 ± 4.76
	Cellular pulmonary	4	5.19 ± 1.49
	Decellularized pulmonary	4	3.28 ± 1.38*
**Circumferential wall samples**	Cellular aortic	8	15.30 ± 7.14
	Decellularized aortic	4	16.00 ± 4.29
	Cellular pulmonary	4	7.94 ± 6.85
	Decellularized pulmonary	4	6.89 ± 1.73
**Radial leaflet samples**	Cellular aortic	8	0.47 ± 0.35
	Decellularized aortic	4	0.84 ± 0.11
	Cellular pulmonary	4	0.36 ± 0.15
	Decellularized pulmonary	4	0.12 ± 0.05*
**Circumferential leaflet samples**	Cellular aortic	8	6.25 ± 0.89
	Decellularized aortic	4	8.56 ± 3.08*
	Cellular pulmonary	4	2.56 ± 2.52
	Decellularized pulmonary	4	3.63 ± 1.10

Data are presented as the mean (*n =* 4 or 8) ± 95% CL. Comparisons between cellular and decellularized samples for the same tissue type were made using Student *t* test.

* indicates significant difference (*p* < 0.05).

## 4 Discussion

In this study, a successful decellularization process developed for porcine aortic and pulmonary valves (Luo *et al*., 2014; Wilcox *et al*., 2005) was transferred to human donor valved roots with a view to manufacturing decellularized cardiac valves for clinical use. Cryopreserved cardiac valved conduits that were unsuitable for clinical application were used since it was impractical to use freshly harvested tissue for the purposes of this study. For porcine valves, the process began by treating the adventitial surface of the arterial wall and ventricular muscle skirt with trypsin to open up the matrix and allow diffusion of decellularization reagents (Luo *et al*., 2014; Wilcox *et al*., 2005). Since human aortic and pulmonary root walls are thinner than porcine, initial studies investigated decellularization of the arterial wall without trypsin and it was found that simple scraping of the outer aortic and pulmonary root wall to disrupt the adventitia was sufficient for decellularization of the full thickness of the arterial walls. The scraped aortic and pulmonary roots were subject to washes in hypotonic buffer and 0.1% (*w*/*v*) SDS in hypotonic buffer to lyse the cells, solubilize the cell membranes and disrupt protein/lipid and protein–protein interactions in the presence of protease inhibitors to prevent enzymatic damage to the structural proteins of the ECM. The roots were then treated with nuclease to cleave the nucleic acids followed by extensive washing in hypertonic buffer and physiological buffer to remove cellular fragments and wash out the SDS.

It was initially found that washing the valved roots using this process was not completely successful with residual DNA (circa 40–50%) remaining within the junctional regions and the leaflets. It was observed that the roots were collapsing in on themselves during the washing process and it was clear that wash solutions were not gaining access to the leaflets and leaflet junctional regions during the decellularization process. Subsequently, all valved roots were decellularized with the lumen maintained open via the use of sutures to suspend the roots from the container. This allowed the wash solutions to move backwards and forwards through the lumen during agitation resulting in successful decellularization of all regions of the valved roots.

The human donor valved roots were decellularized using aseptic technique and sterile solutions throughout. The decision to adopt this approach was based on preliminary studies that showed that treatment of the decellularized valves with peracetic acid ablated collagen IV immunostaining in the basement membrane, and knowledge that irradiation sterilization is not considered appropriate for valve allografts due to the potential for causing deterioration in ECM properties and clinical performance (Helder *et al*., 2016; Sarathchandra *et al*., 2012). Until a reliable sterilization process that does not adversely affect the ECM is demonstrated for biological tissue grafts, it remains acceptable to adopt aseptic production for human tissue products. The wash solutions from several decellularization runs were assessed for microbial contamination by aseptically filtering the solutions onto 0.2‐μm pore sized membranes followed by culture of the membranes on microbiological plates. These were consistently shown to be free of microbial growth.

During the course of these studies the nuclease was changed from Pulmozyme to Benzonase for reasons of economy and utility in a good manufacturing practices manufacturing process. The final concentration of Benzonase to achieve the required reduction in total DNA content (<15 ng/ml) was determined using human donor cryopreserved aortas in a related project. The use of this concentration (10 U/ml) of Benzonase was then directly compared with the use Pulmozyme on porcine pulmonary valved conduits and shown to give slightly better results overall (Pulmozyme: leaflet; 47.9; wall 8.6; junction 14.7; muscle 11.1 ng/mg DNA vs. Benzonase: leaflet 13.4; wall 11.2; junction 8.15; muscle 1.7 ng/mg DNA). It was not deemed necessary to use additional human donor valves to repeat assays, once the change of nuclease had been made.

The Badylak group (Crapo *et al*., 2011) have suggested criteria (DNA content and lack of visible nuclear material in tissue sections stained with H&E or DAPI) by which a tissue can be judged *decellularized* based upon studies in which an *in vivo* constructive remodelling response has been observed. Following decellularization, no evidence of cells, cellular remnants or nuclear material were present in histological sections of the aortic or pulmonary valve tissues and there was no evidence of overt damage to the ECM, as judged by low‐resolution light microscopy. Fenestrations were often observed in the leaflets; however, these are known to be present in cellular valves. The ECM of the decellularized pulmonary arterial wall and leaflets appeared to have a more open, porous structure compared to cellular tissue.

There are limitations of using light microscopy to assess cellular removal and matrix integrity. Alternative imaging methods such as transmission electron microscopy (TEM) can be used to analyse the tissues at higher resolution. However, TEM analysis of the three‐dimensional volume of cardiac valve conduits was considered impractical. In our previous studies of porcine cardiac valved conduits, there was good agreement between the interpretation of light microscopy and TEM analysis of decellularized tissues (Luo *et al*., 2014).

Immunohistochemical analysis was carried out to assess the retention or loss of ECM and membrane proteins. This included analysis for collagen IV, which was used as a marker for the basement membrane, which is believed to be important for endothelialization (Herbst *et al*., 1988) and VWF, which could potentially have the undesirable effect of increasing platelet adhesion to the decellularized matrix if in direct blood contact. Both collagen IV and VWF were present in the decellularized tissues. The retention of collagen IV was considered important since this may encourage endothelialization *in vivo* and mask the underlying thrombogenic matrix. Matrix proteins fibronectin and laminin were assessed since these are important in facilitating cell adhesion and differentiation and both were reduced, but still present postdecellularization, which may indicate minimal impact upon the constructive remodelling of the decellularized tissues *in vivo*. There was an absence of MHC Class 1 immunoreactivity in the decellularized pulmonary valve tissues with only residual staining in the aortic valve tissues, indicating a reduction in the antigenicity of the decellularized tissues. Chondroitin sulfate was assessed since it is the dominant GAG in the proteoglycans in cardiac valve tissue (Rothenburger *et al*., 2002) and showed a qualitative reduction in GAG content postdecellularization. This finding was supported by the quantitative analysis of sulfated sugars (GAGs) which showed that only 25–26% of the original GAGs remained in the valve leaflets following decellularization. Removal of GAGs during decellularization processes incorporating SDS has been reported previously (Luo *et al*., 2014) and was therefore not unexpected. GAGs are a major component of the spongiosa of cardiac valve leaflets, providing hydration and interactions with the fibrous ECM and have been reported to provide a damping mechanism for leaflets during systolic flow (Eckert *et al*., 2013). It is therefore important to determine the effects of the GAG loss on the hydrodynamic performance of the decellularized cardiac valves. Our previous studies of decellularized porcine valved conduits have shown a similar loss of GAGs with no effect on the valve performance in hydrodynamic tests (Luo *et al*., 2014). The loss of GAGs and cellular elements in the decellularized valve leaflet tissues would account for the apparent increase in collagen content per weight of dried tissue compared to cellular tissue.

Importantly, the decellularization process had essentially removed all but residual DNA from the tissues with <10 ng/mg for all tissue regions with the exception of the pulmonary valve leaflets. It should be noted that estimation of the DNA content of successfully decellularized tissues is problematic. In order to extract measurable amounts of DNA, the initial mass of the tissue required for extraction is 10‐fold higher than that required for cellular tissue. For the decellularized pulmonary valve leaflets the total mass of tissue from each of the decellularized valves was only 30–100 mg and the levels of DNA extracted were at the limits of detection of the nano‐spectrophotomer. Hence the data for the estimation of DNA content of decellularized pulmonary leaflets were not reliable and this was a limitation of the study.

It is of interest to compare our decellularization process to others applied to human donor cardiac valves that have been used clinically. The Synergraft process uses hypotonic cell lysis, nuclease digestion and isotonic washes but does not incorporate a detergent wash (Gerson *et al*., 2012; O’Brien *et al*., 1999; Zehr *et al*., 2005). The process has, however, been reported to reduce MHC immunoreactivity markedly, indicating reduction in membrane proteins (Elkins *et al*., 2001b). The process described by da Costa et al. (2010) uses 0.1% (*w*/*v*) SDS and washing in isotonic Ringer lactate, but does not mention the use of nuclease and the process described by the Haverich group (Cebotari *et al*., 2011; Neumann *et al*., 2014) incorporates a harsher detergent treatment (0.5% *w*/*v* SDS plus 0.5% *w*/*v* sodium deoxycholate) but again does not appear to incorporate a nuclease treatment (Cebotari *et al*., 2011). Despite the fact that these decellularized human donor cardiac valves have been implanted into patients now for several years, details of the DNA levels in these decellularized human donor valve allografts is lacking. This is surprising given that DNA levels are a good indicator of the degree of cell removal (Crapo *et al*., 2011) and the potential role of residual cellular DNA in calcification of tissue heart valve substitutes (Schoen and Levy, 2005). To our knowledge this is first report of a decellularization process that removes over 97% of the DNA from the junctional region of human aortic and pulmonary valve conduits. In our experience with porcine valves these regions are the most resistant to decellularization.

The decellularized tissues were not toxic to cells *in vitro* using standard extract and cytotoxicity tests. Hence studies progressed to evaluate the biocompatibility of the decellularized tissues *in vivo* using an established mouse subcutaneous implant model which has been previously shown to be an excellent indicator of the propensity of tissues for calcification (Mirsadraee *et al*., 2007). Initial studies indicated that the decellularized aortic and pulmonary leaflet and wall tissues showed evidence of tissue integration and recolonization by host cells; however, a proportion of the samples revealed evidence of foci of calcification. Further study revealed the presence of calcium deposits in the decellularized tissues prior to implantation in the mouse model and this explained the result. Since the tissues investigated were from donors with a mean age of 58, the study was repeated using heart valves from younger donors. The repeat study indicated biocompatibility of the decellularized aortic and pulmonary wall and leaflet tissues in the mouse model in comparison to encapsulation and calcification of the cellular tissues from the arterial walls of the same donor valves. Interestingly, there was little evidence of a specific immune component to either the cellular or decellularized tissues with the response to both types of tissue orchestrated by macrophages. The role of macrophages in the regeneration of acellular biological scaffolds has been well documented by ourselves and others (Brown *et al*., 2012; Paniagua Gutierrez *et al*., 2015) and hence this was not unexpected. There was clearly a different outcome for the cellular compared to the decellularized tissue in the mouse model with limited cellular infiltration into the cellular tissue together with dystrophic calcification presumably due to presence of dead cells and cellular infiltration into the central areas of the decellularized tissue in the absence of tissue degradation or necrosis. It is important to note that the mouse model, although useful for assessment of biocompatibility is not a functional model for cardiac valve tissue.

Uniaxial tensile testing of the cellular and decellularized aortic and pulmonary valve tissues was used to determine the maximum load to failure. Here we chose to analyse the mechanics based on force rather than stress, to avoid recording changes due to changes in tissue thickness and to make the results more relevant to the clinical application. This showed a high degree of variation in both the cellular and decellularized tissues, as might be expected with human donor valves. Overall, the data indicated that the decellularization process produced moderate effects on the strength of the valve arterial and leaflet tissues. A full biomechanical and haemodynamic performance analysis of the decellularized valves has been undertaken and will be reported elsewhere.

There are obvious limitations to this study, not least the low number of replicate valves used for the various analyses and the high degree of variation in the quantitative data presented. However, the study used human donor tissue, which unlike porcine tissue is not readily available and hence the high degree of variability reflected the variation in the donated tissues in terms of age, sex and also lifestyle of the individual donors.

In conclusion, a process for the decellularization of human donor aortic and pulmonary cardiac valved conduits has been developed which has been shown to produce biocompatible grafts with virtually no DNA and acceptable bulk biomechanical properties. The process will now be translated to a good manufacturing practices‐compatible process for donor valves. However, valves from younger donors may be preferable for processing in order to avoid premature calcification.

## Statement of on potential conflicts of interest

At the time that this study was conducted, HB was an employee of Tissue Regenix Group Plc. EI and JF are advisors to Tissue Regenix Group Plc and hold stock in the company.

## Sponsors

1. WELMEC a Centre of Excellence in Medical Engineering funded by the Wellcome Trust and EPSRC, under grant number WT 088908/Z/09/Z.
2. N8; Regener8 Collaborative R&D Project; Acellular allogeneic cardiac valves.
